# Prenatal Diagnosis and Monitoring of Type III Congenital Pulmonary Airway Malformation: A Case Report

**DOI:** 10.7759/cureus.109508

**Published:** 2026-05-23

**Authors:** Emma Huber, Ariana Tiberi, Wanda I Torres

**Affiliations:** 1 Obstetrics and Gynecology, Nova Southeastern University Dr. Kiran C. Patel College of Osteopathic Medicine, Clearwater, USA; 2 Obstetrics and Gynecology, Suncoast Women's Care, Trinity, USA

**Keywords:** congenital pulmonary airway malformation, congenital thoracic malformation, cpam, fetal intervention, lung development, neonatal management, prenatal diagnosis

## Abstract

Congenital pulmonary airway malformation (CPAM) is a rare congenital lung anomaly resulting from abnormal bronchial development and disorganized respiratory structures, producing nonfunctional pulmonary lesions. Although uncommon, CPAM is the most frequently diagnosed congenital lung malformation. Prenatal identification allows for risk stratification and anticipatory management. Here we present a case of a fetus prenatally diagnosed with a type III CPAM during routine obstetric ultrasonography. Weekly ultrasonography was performed to monitor lesion progression and evaluate for complications, including mediastinal shift and hydrops fetalis. Multidisciplinary prenatal surveillance guided management during pregnancy. The pregnancy course, delivery planning, and neonatal outcomes are presented.

## Introduction

Congenital pulmonary airway malformation (CPAM) is a rare developmental anomaly of fetal lung tissue during embryogenesis, resulting in bronchioles that do not participate in gas exchange. Although congenital lung anomalies are rare, CPAM is the most common developmental lung anomaly. The pathogenesis of CPAM is not fully understood; however, current evidence suggests an imbalance between cellular proliferation and apoptosis, leading to arrested lung maturation. This process may be driven by the overexpression of growth factors, such as KGF, PDGF-B, and GDNF. Additionally, some studies have identified potential associations with mutations in the HOXB5, LRP2, and TP53 genes [1,2]. Despite these findings, CPAM is generally considered to occur sporadically and has not been linked to maternal or paternal age, ethnicity, or other maternal risk factors. Without severe complications, such as hydrops fetalis or significant pulmonary hypoplasia, the overall prognosis is generally favorable [3]. Prenatal diagnosis allows for adequate risk stratification and longitudinal monitoring to guide management and delivery planning [4]. 

The CPAM volume ratio (CVR) is a metric used to monitor the lesion over time and predict the risk of fetal hydrops [5]. The CVR is calculated using the following equation: CVR = (length x width x height x 0.52)/head circumference. A CVR greater than 1.6 cm^2^ is associated with 75% risk of developing fetal hydrops, whereas a CVR less than 1.6 cm^2^ is associated with 17% risk [6,7]. Importantly, CVR trend over time is often more clinically informative than a single measurement. A decreasing CVR trend implies the lesion is not growing significantly relative to fetal growth over time, and it may regress. Conversely, a rapidly increasing CVR warrants closer surveillance due to a higher risk of mediastinal compression, cardiovascular compromise, and progression to hydrops fetalis [5,6]. 

Most CPAMs peak growth is between 25 and 26 weeks’ gestation, after which lesions commonly stabilize or decrease in size relative to the fetus growth during the third trimester [3]. Serial ultrasonography during this period is therefore critical, as changes in CVR trajectory can influence management decisions, including the consideration of corticosteroids to assist lung maturity, commonly antenatal betamethasone. In the absence of further structural abnormalities, routine invasive testing for fetal aneuploidy is not recommended [4]. 

Prognosis becomes more variable in cases complicated by fetal hydrops or pulmonary hypoplasia, where the large or rapidly expanding lesion may compress mediastinal structures, resulting in impaired blood flow or lung development. Isolated CPAMs are associated with the most favorable outcomes [3,7,8]. Based on severity, management strategies may include maternal corticosteroid administration, thoracocentesis, thoracoamniotic shunt placement, open fetal surgery, or delivery via an ex utero intrapartum treatment (EXIT) procedure in select cases [6]. Postnatal surgical excision is generally curative and is associated with excellent long-term outcomes when adequate lung development is present. If the CVR is greater than 1.6 cm^2^, then without surgical intervention, affected infants may be at an increased risk for recurrent pulmonary infections and rare malignant transformation. These include pleuropulmonary blastoma and bronchioloalveolar carcinoma, which are uncommon but most likely with a type IV CPAM [3,9].

CPAMs are classified into subtypes based on cyst size and histopathologic features. Type I CPAM, the most common subtype, consists of large cysts greater than 2 cm lined by respiratory epithelium and carries the most favorable prognosis. Type II CPAM consists of multiple small cysts less than 2 cm and is frequently associated with other congenital anomalies, including cardiac, renal, and diaphragm defects. Type III CPAM consists of a microcystic lesion that appears uniformly echogenic and solid on ultrasound. This type may cause mediastinal shift and fetal hydrops, and therefore is associated with the poorest prognosis [8]. The unique case presented here describes a fetus diagnosed prenatally with a type III CPAM, including the serial ultrasounds and prognosis. 

## Case presentation

A female neonate was delivered at 36 weeks and five days of gestation to a 32-year-old G2P0101 Caucasian female. Prenatal care was notable for the incidental detection of a fetal lung lesion during routine obstetric ultrasonography. The patient had no significant medical comorbidities, and the pregnancy had been uncomplicated before diagnosis.

**Figure 1:**
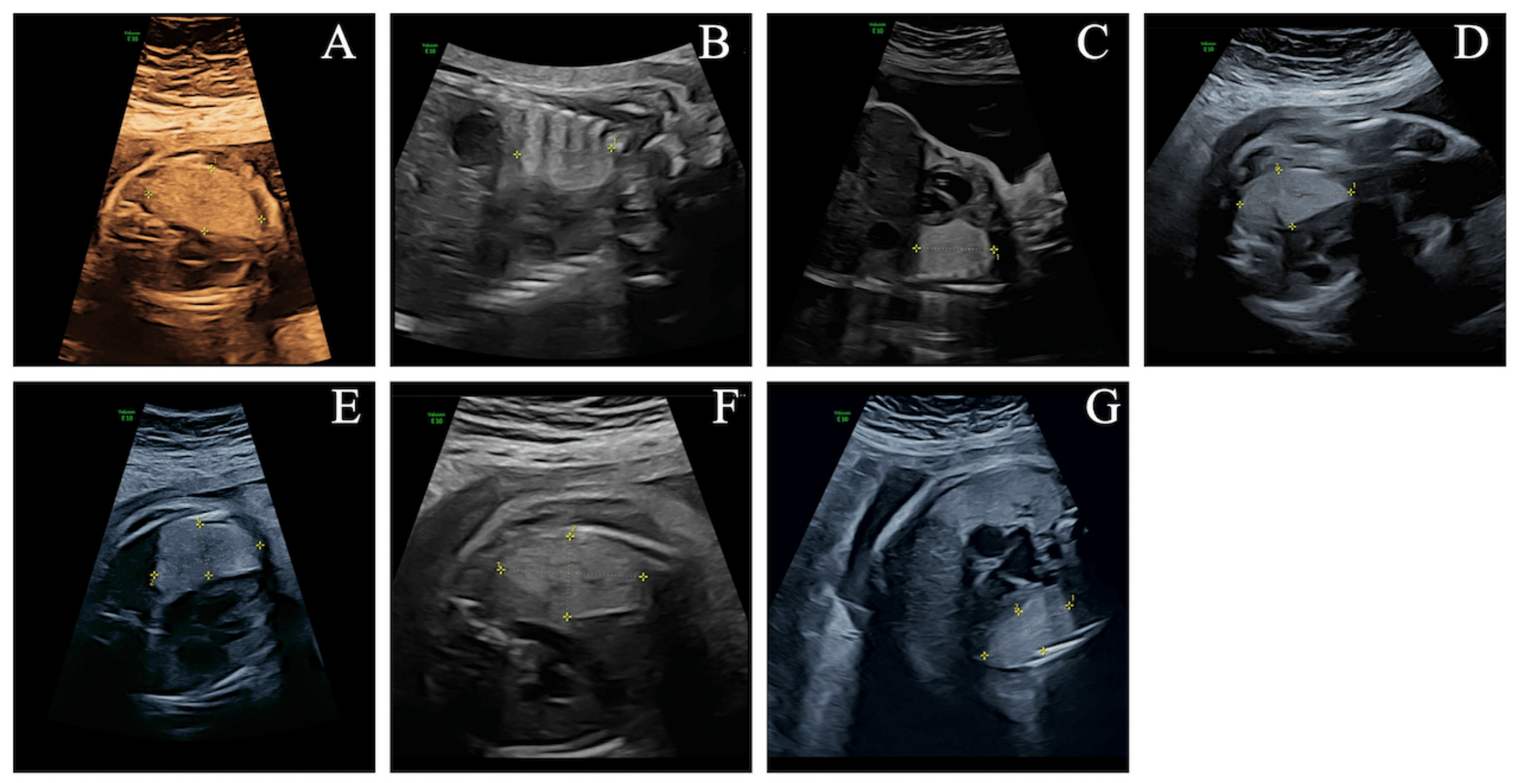
CPAM ultrasounds at various gestational ages A: Ultrasound demonstrating left-sided CPAM with CVR = 0.48 cm^2^ at 23 weeks of gestation. There is no evidence of fetal hydrops. Image obtained during Maternal Fetal Medicine evaluation at Florida Perinatal Associates, with patient consent. B: Ultrasound demonstrating left-sided CPAM with CVR = 0.51 cm^2^ at 24 weeks of gestation. There is no evidence of fetal hydrops. Image obtained during Maternal Fetal Medicine evaluation at Florida Perinatal Associates, with patient consent. C: Ultrasound demonstrating left-sided CPAM with CVR = 0.45 cm^2^ at 25 weeks of gestation. There is no evidence of fetal hydrops. Image obtained during routine Maternal Fetal Medicine evaluation at Florida Perinatal Associates, with patient consent. D: Ultrasound demonstrating left-sided CPAM with CVR = 0.63 cm^2^ at 26 weeks of gestation. There is no evidence of fetal hydrops. Image obtained during routine Maternal Fetal Medicine evaluation at Florida Perinatal Associates, with patient consent. E: Ultrasound demonstrating left-sided CPAM with CVR = 0.31 cm^2^ at 27 weeks of gestation. There is no evidence of fetal hydrops. Image obtained during routine Maternal Fetal Medicine evaluation at Florida Perinatal Associates, with patient consent. F: Ultrasound demonstrating left-sided CPAM with CVR = 0.33 cm^2^ at 28 weeks of gestation. There is no evidence of fetal hydrops. Image obtained during routine Maternal Fetal Medicine evaluation at Florida Perinatal Associates, with patient consent. G: Ultrasound demonstrating left-sided CPAM with CVR = 0.27 cm^2^ at 30 weeks of gestation. There is no evidence of fetal hydrops. Image obtained during routine Maternal Fetal Medicine evaluation at Florida Perinatal Associates, with patient consent.

The fetal lung malformation was first detected at 18 weeks and 0 days’ gestation during a Maternal Fetal Medicine anatomic ultrasound scan. According to the Maternal Fetal Medicine reports, it presented as a homogenous echogenic mass in the left thorax, which appeared to receive its blood supply from the pulmonary artery. This is consistent with a congenital pulmonary airway malformation. It is composed of very small cysts with a homogenous echogenic appearance, consistent with a type III CPAM. The mass was found to be 2.84 x 1.19 x 2.47 cm. The CVR was 0.27 cm^2^, which indicated low risk for the development of fetal hydrops. The normal appearing lung was superior to the echogenic mass. The fetal heart was mildly displaced to the right. The cardiac axis was normal. There was no evidence of pleural effusion. This ultrasound was not provided by Florida Perinatal Associates. As the fetus developed, serial weekly ultrasounds were completed to monitor size, CVR, and for evidence of hydrops (Figure 1). All sonographic images were obtained during clinical care and provided by Dr. Jeffery Angel, MD, at Florida Perinatal Associates. *(If an ultrasound is not listed as a figure for a specific gestational age, the Maternal Fetal Medicine report did not include it.)*

Weekly ultrasounds were performed to monitor lesion size, measure the CVR pattern, and detect signs of cardiopulmonary compromise (Figure 1). According to the Maternal Fetal Medicine reports, the lesion demonstrated gradual growth, reaching a peak size at 21 weeks and 0 days’ gestation with a maximum CVR of 0.74 cm^2^. This sonogram was not provided by Florida Perinatal Associates. Thereafter, serial ultrasounds showed stabilization and regression of the lesion relative to fetal growth. At the time of delivery, the CPAM had decreased in size to near immeasurable. Weekly ultrasound measurements obtained by Maternal Fetal Medicine are summarized in Table 1 *(If a measurement is not listed, the Maternal Fetal Medicine report did not include it).*

**Table 1:** Serial sonogram measurements of CPAM throughout pregnancy CPAM: congenital pulmonary airway malformation; CVR: CPAM volume ratio; MVP: maximum vertical pocket

Gestational age	L x W x H (cm)	CVR (cm^2^)	Amniotic MVP
18 wk 0 d	2.84 x 2.47 x 1.19	0.27	Not available
19 wk 0 d	Not available	0.21	Not available
20 wk 0 d	Not available	0.20	4.56
21 wk 0 d	3.91 x 3.77 x 1.87	0.74	5.20
22 wk 0 d	3.89 x 2.39 x 1.97	0.45	4.10
23 wk 0 d	Not available	0.48	4.51
24 wk 0 d	4.29 x 2.97 x 1.95	0.51	5.27
25 wk 0 d	4.00 x 3.08 x 1.71	0.45	5.10
26 wk 0 d	4.12 x 3.28 x 2.13	0.63	4.56
27 wk 0 d	2.48 x 3.55 x 1.67	0.31	5.12
28 wk 0 d	2.58 x 3.33 x 1.88	0.33	3.48
Maternal Fetal Medicine changed the measurements from weekly to every two weeks.			
30 wk 0 d	0.68 x 0.56 x 0.52	0.27	4.14
32 wk 0 d	0.71 x 0.88 x 0.78	Near immeasurable	4.16
34 wk 5 d	0.12 x 0.85 x 0.95	Near immeasurable	3.96
36 wk 5 d	Delivery		

The remainder of the prenatal course was largely unremarkable until 32 weeks and five days’ gestation, when the patient presented with vaginal bleeding and was admitted for inpatient monitoring. Ultrasound evaluation demonstrated a complete placenta previa. The patient received a two-dose course of antenatal betamethasone for fetal lung maturation. After 48 hours of observation without recurrent bleeding, she was discharged with plans for scheduled cesarean delivery at 37 weeks’ gestation.

At 36 weeks and five days’ gestation, the patient re-presented with vaginal bleeding and underwent an uncomplicated cesarean delivery. A female neonate weighing 3,118 g (6 lb 14 oz) was delivered in cephalic presentation with Apgar scores of 8 and 8 at one and five minutes, respectively. The placenta was manually extracted without complication. Given the preterm delivery, the neonate was admitted to the neonatal intensive care unit for observation and supplemental oxygen as needed. The neonatal course was uncomplicated, and the infant was discharged home with the mother. At the one-week postpartum follow-up visit, both mother and infant were clinically well, with no reported respiratory concerns in the neonate.

## Discussion

This case highlights the prenatal history, surveillance, and favorable outcome of a prenatally diagnosed type III CPAM with a consistently low, and regressing CVR. Although type III CPAMs are traditionally associated with poorer prognoses due to their solid, microcystic composition and tendency for complications, such as mediastinal compression and hydrops fetalis, this case demonstrates that consistent weekly monitoring of CVR trends can effectively guide expectant management and avoid unnecessary fetal intervention.

The mother’s history was non-significant with the absence of medical conditions commonly known to complicate pregnancy, including tobacco or substance use, diabetes, hypertension, autoimmune disease and blood disorders. Maternal age, ethnicity, reproductive history, and health status are consistent with the existing literature demonstrating that CPAMs typically occur sporadically and are not strongly associated with maternal characteristics or inherited genetic conditions [3]. Her obstetric history was significant for a previous preterm delivery due to placenta previa that subsequently resolved. Previous placenta previa is a risk factor for recurrent placenta previa, as seen in this pregnancy resulting in cesarean delivery.

Prenatal ultrasonography is the gold standard imaging for CPAM diagnosis. It is typically found incidentally during the second-trimester. In this case, the lesion was identified at 18 weeks’ gestation as a homogeneous echogenic mass in the left thorax with blood supply by the pulmonary artery, consistent with a type III CPAM. Early diagnosis in this case allowed for timely referral to Maternal Fetal Medicine and initiation of weekly ultrasound surveillance. Importantly, no associated structural anomalies were identified, supporting the existing evidence that isolated CPAMs carry a more favorable prognosis.

CPAM should be differentiated from other congenital lung conditions. Most significant is bronchopulmonary sequestration, a nonfunctional lung tissue supplied by systemic circulation. Bronchopulmonary sequestration is frequently present in the lower lobe of the lung and typically appears as a homogenous or heterogeneous solid mass on imaging. The most crucial feature to distinguish between bronchopulmonary sequestration and CPAM is the blood supply. Bronchopulmonary sequestration receives its blood supply from the systemic arteries, most commonly the thoracic aorta, whereas CPAM receives its blood supply from the pulmonary artery [10]. Type III CPAM is the most difficult to differentiate from bronchopulmonary sequestration. However, in this patient, the blood supply was noted to be from the pulmonary artery by Maternal Fetal Medicine at 18 weeks of gestation. 

CVR trend is accepted as a validated prognostic tool for stratifying the risk of fetal hydrops and monitoring lesion behavior over time. A CVR threshold of 1.6 cm^2^ is widely cited, with values above this level conferring a substantially increased risk of hydrops, while values below 1.6 cm^2^ are associated with lower risk [7,11]. In this case, the initial CVR was 0.27 cm^2^, indicating minimal risk. Although the lesion demonstrated growth with a peak CVR of 0.74 cm^2^ at 21 weeks’ gestation, the CVR remained well below the high-risk threshold of 1.6 cm^2^ throughout pregnancy. This demonstrates that CVR trajectory is often more clinically meaningful than absolute lesion size or a single CVR measurement. Serial weekly and eventually biweekly ultrasounds allowed for timely reassurance and avoidance of fetal interventions, such as thoracoamniotic shunting or open fetal surgery, which are reserved for cases complicated by hydrops or progressive cardiopulmonary compromise [3,4]. A two-dose betamethasone series was administered at 32 weeks five days of gestation in this pregnancy for obstetric complications related to vaginal bleeding rather than as a consequence of the CPAM. In this patient, the lesion had already demonstrated spontaneous regression before steroids, which is consistent with the literature stating that a substantial proportion of CPAMs stabilize or involute during the third trimester [5]. 

Delivery planning in CPAM pregnancies is individualized per patient based on the lesion size, CVR progression, and anticipated neonatal respiratory compromise. If the CPAM is of significant size at the time of delivery, then the baby should be delivered at a level 4 center with the availability of pediatric surgery and high-level NICU. Smaller lesions can deliver at a level 3 center, typically with MRI performed after delivery and referral to a pediatric surgeon. Because the lesion had regressed to near nonvisualization by late gestation and there was no evidence of hydrops or cardiopulmonary compromise, delivery was scheduled for 37 weeks of gestation. However, delivery at 36 weeks and five days occurred for maternal placenta previa rather than fetal concerns. Neonatal outcomes were favorable, with a brief NICU stay for observation only due to prenatal delivery and not for any respiratory complications attributable to the CPAM. Additionally, because this patient had a CVR less than 1.6 cm^2^ near the time of delivery, a tertiary delivery center was not involved. 

This case illustrates the importance of multidisciplinary care involving obstetrics, Maternal Fetal Medicine, neonatology, and pediatric surgery. While postnatal surgical excision is generally recommended for CPAMs due to the risks of infection and rare malignant transformation, timing and necessity depend on postnatal imaging and clinical course. Continued outpatient follow-up and postnatal imaging will be essential to guide long-term management in this patient. Even type III CPAMs, traditionally considered higher risk, can have excellent outcomes when the CVR remains under 1.6 cm^2^ and demonstrates a regressive trend. Careful interpretation of serial CVR measurements, rather than reliance on lesion type alone, is critical in guiding management, counseling families, and avoiding unnecessary fetal intervention.

## Conclusions

This case report highlights the importance of early prenatal diagnosis and close surveillance of a type III CPAM. A coordinated, multidisciplinary approach is essential to optimize fetal outcomes and guide delivery planning in pregnancies complicated by fetal lung malformations. Importantly, this case demonstrates that type III CPAMs do not all result in poor or fatal outcomes and that, without fetal hydrops and cardiopulmonary compromise, favorable neonatal outcomes can be achieved with careful monitoring and appropriate perinatal care.
